# Localised relative scotoma in cuticular drusen

**DOI:** 10.1007/s00417-022-05570-4

**Published:** 2022-02-07

**Authors:** Jason Charng, Chandrakumar Balaratnasingam, Mary S. Attia, Rachael C. Heath Jeffery, Fred K. Chen

**Affiliations:** 1 Centre of Ophthalmology and Visual Science (Incorporating Lions Eye Institute), University of Western Australia, 2 Verdun Street, Perth, WA 6009 Australia; 2 Department of Optometry, School of Allied Health, University of Western Australia, Perth, WA Australia; 3 Department of Ophthalmology, Sir Charles Gairdner Hospital, Perth, WA Australia; 4 Department of Ophthalmology, Royal Perth Hospital, Perth, WA Australia; 5 Ophthalmology, Department of Surgery, University of Melbourne, Melbourne, Victoria Australia

**Keywords:** Basal laminar drusen, Microperimetry, Retinal sensitivity, Normative reference

## Abstract

**Purpose:**

To investigate retinal sensitivity changes in eyes with pure cuticular drusen.

**Methods:**

Multimodal imaging and microperimetry (37-loci grid) data were examined retrospectively to evaluate functional changes in eyes with pure cuticular drusen. Mean sensitivity in the cuticular drusen cohort was compared to age-matched normals. An age- and loci-specific normative reference was created to analyse localised sensitivity deviation.

**Results:**

The mean number loci with relative scotoma in the cuticular drusen cohort (*n* = 27, mean [SD] age: 48.5 [12.4] years) referenced to normal eyes (*n* = 80, 53.5 [14.6] years) was 5.5 (95% confidence interval 3.0 to 8.1). However, mean sensitivity was not statistically different to the age-matched normal cohort (95% CI, − 2.3 to + 3.4 dB). The 37-loci grid was stratified into three rings of the approximately same number of loci, and the percentage of cuticular drusen eyes with pointwise deviation was significantly lower in the inner compared to the middle ring (12.3 [5.3]% vs. 17.3 [5.1]%, *p* < 0.05).

**Conclusions:**

Eyes with cuticular drusen demonstrated relative scotoma, but mean sensitivity was not affected. Pointwise sensitivity provides a more robust measure of retinal sensitivity than mean sensitivity in cuticular drusen and should be assessed both in the clinic and in future clinical trials.

**Supplementary Information:**

The online version contains supplementary material available at 10.1007/s00417-022-05570-4.


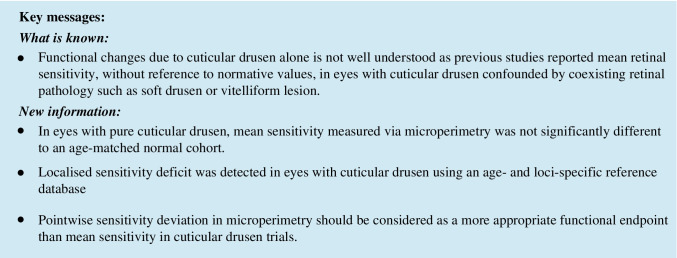


## Introduction

Cuticular drusen are recognised as a distinct phenotype in the spectrum of age-related macular degeneration (AMD) that has a resemblance to hard drusen histologically and yet confers a unique risk of progression to sight-threatening complications similar to but potentially decades earlier than, soft drusen [1]. Multimodal imaging has localised cuticular drusen to the subretinal pigmented epithelium (RPE)-basal laminar compartment [1–3]. Structurally, cuticular drusen are indistinguishable from hard drusen, consisting of dense, hyalinised content and appear as small, well-demarcated and steep-sloped elevations [1]. While global RPE dysfunction has been suggested in cuticular drusen [4], histologically, there is only attenuation of the RPE immediately overlying the cuticular druse [1, 5]. Importantly, the impact on retinal function due to cuticular drusen has not been investigated in detail.

An earlier study used automated perimetry to demonstrate reduced central retinal sensitivity in cases of cuticular drusen complicated by vitelliform macular detachment [6]. However, without fundus tracking and overlay of sensitivity map on fundus images, it was not possible to determine if the retinal sensitivity reduction was due to the vitelliform lesion alone or in combination with the surrounding cuticular drusen. Recently, it has been shown that mesopic microperimetry mean sensitivity is decreased in eyes with cuticular drusen (− 2.1 dB, *n* = 9, 57.6 ± 8.8 years) compared to a small control cohort (*n* = 20, 61.7 ± 12.4 years) [7]. Note that the study recruited patients with intermediate AMD, hence the impact of coexisting soft drusen and hyper-pigmentation may have confounded the results. Furthermore, the comparison was not controlled for age and spatial distribution given the normative values for microperimetry sensitivity changes with age [8, 9] and retinal eccentricity [8, 10] in healthy eyes. Therefore, small but significant functional changes may be masked by analysis of mean sensitivities alone.

In this study, we define via multimodal imaging a cohort with cuticular drusen with no sight-threatening complications such as vitelliform deposits, geographic atrophy, or choroidal neovascularization. Microperimetry was utilised to assess the effect of cuticular drusen alone on retinal sensitivity.

## Methods

This is a retrospective study that was conducted in accordance to the tenets of the Declaration of Helsinki. The study was approved by the human research ethics committee of The University of Western Australia (2021/ET000151) and written informed consent was obtained from each subject for their imaging and MAIA data to be used for research purposes.

### Selection of study eyes

#### Healthy cohort

The MAIA (Macular Integrity Assessment; CenterVue, Padova, Italy) microperimetry database was searched for eligible subjects. Inclusion criteria were best-corrected visual acuity of 20/25 or better, normal retina, and optic nerve head based on fundus examination and normal retinal scans from optical coherence tomography (Spectralis HRA + OCT, Heidelberg Engineering, Heidelberg, Germany) and fundus autofluorescence (California, Optos plc, Dunfermline, UK), as confirmed by the senior author (FKC). Exclusion criteria were previous eye surgery (e.g. cataract extraction, retinal laser), diabetes mellitus, amblyopia, or history of medication use that may potentially affect photoreceptor function (e.g. hydroxychloroquine, antipsychotics, tamoxifen). Some subjects are healthy controls recruited into the Western Australia Retinal Degeneration study and others were routine clinic patients with unilateral retinal disease. In patients with bilateral eligible eyes, MAIA data from the right eye were chosen for analysis. In patients with unilateral eye disease, the non-diseased eye was chosen if eligible. The first MAIA test performed by each subject was analysed.

#### Cuticular drusen cohort

The clinical database was interrogated for patients with a clinical diagnosis of cuticular drusen. The autofluorescence (short wavelength [488 nm excitation] and near-infrared wavelength [787 nm excitation]) and optical coherence tomography (OCT) images of these patients were reviewed by two retinal specialists to confirm the clinical diagnosis (CB, FKC). The right eye was chosen as the study eye if both eyes were eligible. The same exclusion criteria as the healthy cohort were applied to the cuticular drusen patients except for those with a history of cataract surgery. If cataract surgery was performed at least 6 months prior to the first MAIA test, and there was no clinical complication from the procedure (i.e. posterior capsular opacification, macular oedema), then the eye was deemed eligible for the study. Eyes with vitelliform deposits, geographic atrophy, choroidal neovascularization, typical soft drusen, or drusenoid or serous pigment epithelial detachment were excluded.

### Microperimetry testing protocol

All MAIA testing was performed by trained ophthalmic assistants in a single institution. Multimodal imaging was performed after MAIA testing followed by a retinal examination by a retinal specialist (FKC).

**Fig. 1:**
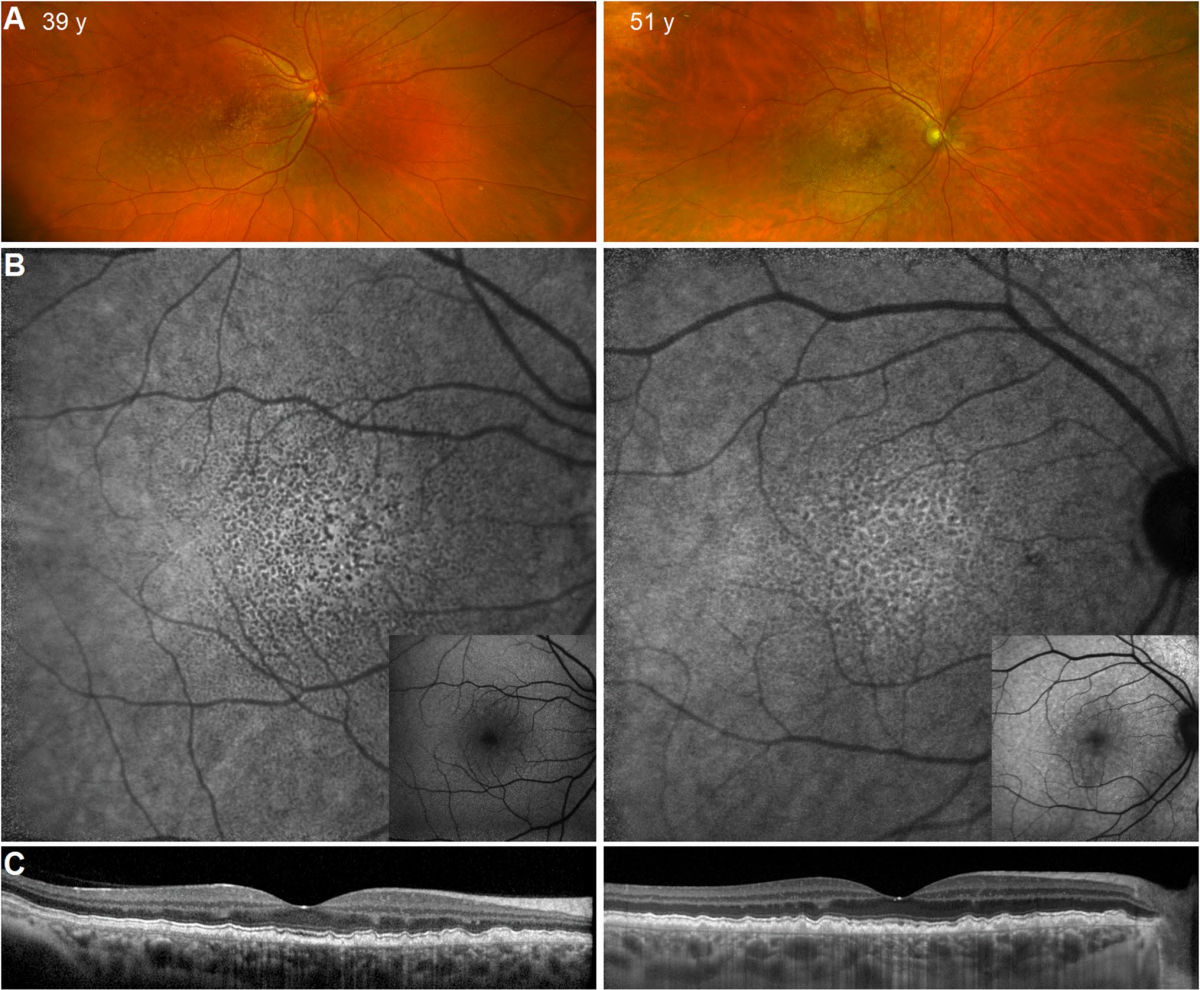
Cuticular drusen multimodal imaging. **A** Pseudocolor imaging of 2 patients with cuticular drusen (left, 39 years; right, 51 years). **B** Near-infrared autofluorescence imaging of the macular region of the same eyes from panel A (inset shows corresponding short-wavelength autofluorescence image). **C** Corresponding horizontal foveal OCT scans from the same eyes

The MAIA test grid samples retinal sensitivity from the fovea to the perifovea. It comprises 37 loci arranged in an annular pattern [11]. The test loci are located at 0°, 2.3°, 4°, and 6° eccentricity from the fovea (Fig. 1A, B). Goldman III achromatic stimuli with 200 ms duration were presented on a dim white background (1.27 cd.m^−2^) one at a time. The dynamic range of the differential stimulus luminance is 0.08 to 317.04 cd/m^2^, which corresponds to 36 to 0 dB. A 4–2 staircase test strategy was chosen. For each subject, to be included in the analysis, 63% bivariate contour ellipse area was less than 5^02^.

### Statistical analysis

Raw pointwise data was extracted from the MAIA device with left eye data transformed to right eye orientation. In the MAIA software, a locus that could not be detected by the patient at the highest luminance was signified with − 1 dB, and this terminology was kept for analysis.

Quantile regression was utilised to establish loci- and age-specific reference from healthy eyes as previously reported [8]. In brief, at each locus, retinal sensitivity was plotted against age, and quantile regression was utilised to establish the 95% prediction interval [12, 13]. Hence for each cuticular drusen patient, at each locus, sensitivity below the 2.5th percentile line would be defined as a below-normal retinal function. This analysis method provides age- and location-adjusted sensitivity for each retinal locus. R v.4.0.0 (R Core Team, R: A language and environment for statistical computing. R Foundation for Statistical Computing, Vienna, Austria. URL https://www.R-project.org/) was used to perform all statistical analysis [14], and quantile regression was conducted with the quantreg package v5.38 [15].

The pointwise deviation was calculated at each locus by subtracting the age-matched sensitivity value calculated from the median (i.e. 50th percentile) line at the same age from the patient’s sensitivity at the locus. A negative value indicates that the patient’s actual measured sensitivity was less than the age- and location-adjusted sensitivity. For each eye, the pointwise sensitivity deviation at all loci was then averaged across the whole grid (i.e. mean sensitivity deviation).

Group data are summarised by average, standard deviation (SD), and 95% confidence interval (CI) where appropriate. Welch’s two sample *t*-test, which is robust comparing groups of unequal sample size, was utilised to compare the difference of the means between the normal and the cuticular drusen cohort. A linear equation was used to examine the effect of age on mean sensitivity in both normal and cuticular drusen cohorts. The equation parameters, *R*^2^ and *p*-value of the linear fits for each cohort were calculated.

## Results

### Patient demographics

Eighty eyes from 80 participants were included in the healthy cohort. The mean (SD) age was 53.5 (14.6) years, and the female:male ratio was 11:9. Among the 80 eyes, 28 had bilateral normal eyes, and the rest had unilateral eye disease (Supplementary Table S1). Twenty-seven eyes from 27 patients were included in the cuticular drusen cohort (Supplementary Table S2). The mean (SD) age was 48.5 (12.4) years, and the female:male ratio was 18:9. All patients with cuticular drusen were phakic, 1 has a clinical diagnosis of cerulean cataract and 4 with mild nuclear sclerosis cataract. The two cohorts were similar in age (*t*-test, *p* = 0.09).

### Cross-sectional retinal sensitivity in cuticular drusen

Multimodal imaging from two representative eyes with cuticular drusen are shown in Fig. 1. Pseudocolour images show pale lesions clustered on the macula in both eyes (Fig. 1A, left: 39 years, right: 51 years). Near-infrared autofluorescence images demonstrate scattered hypoautofluorescent dots with surrounding hyperautofluorescence (Fig. 1B), with unremarkable short-wavelength autofluorescence (inset). The corresponding horizontal OCTs through the fovea confirms numerous small sub-RPE elevations (Fig. 1C).

**Fig. 2:**
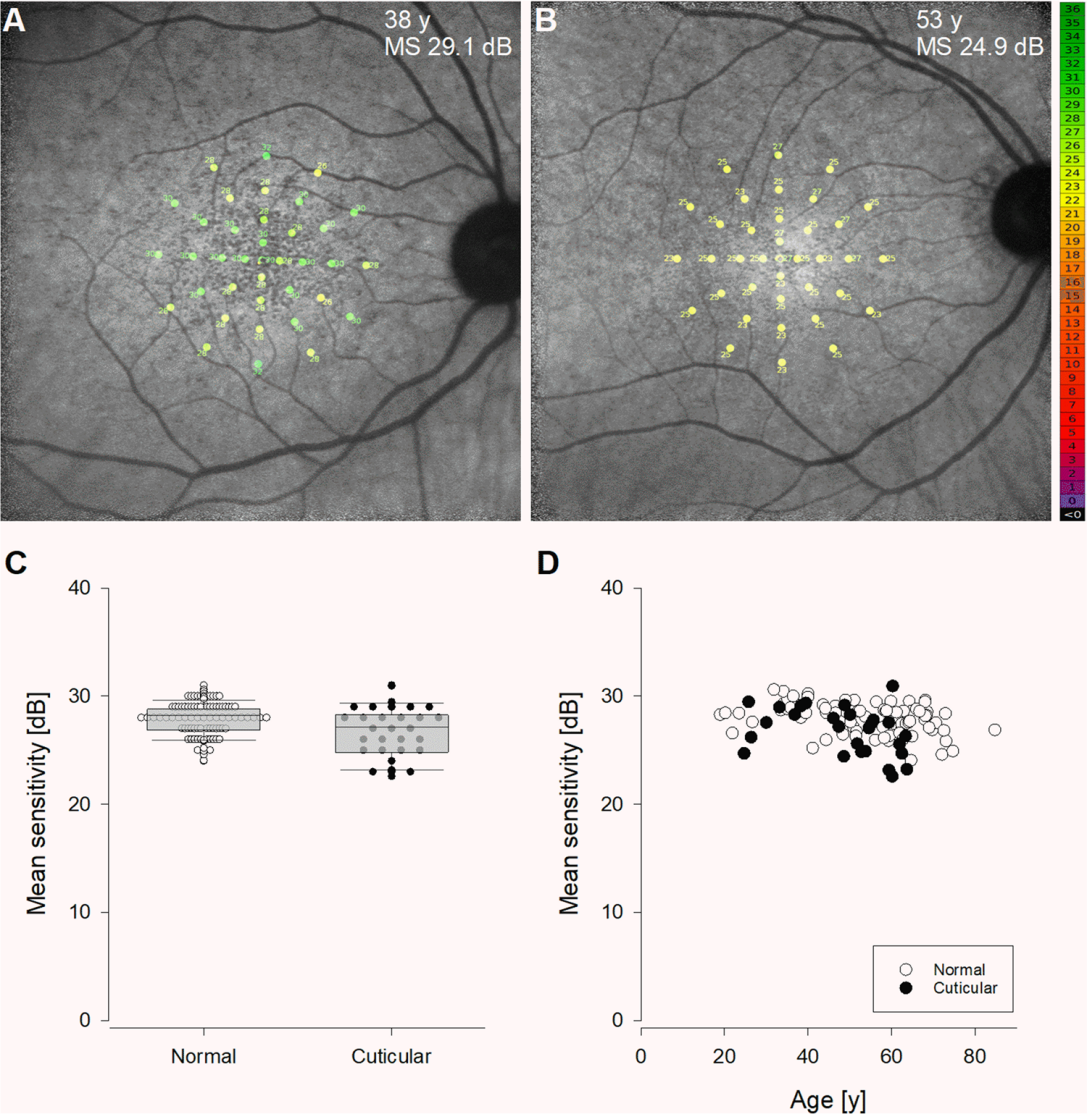
Cross-sectional retinal sensitivity in cuticular drusen. **A**, **B** The MAIA test grid is overlaid on the near-infrared autofluorescence images of a 38-year-old (**A**) and a 53-year-old eye with cuticular drusen. Age and mean sensitivity (MS) are shown in the upper-right corner. The colour scale bar at the right refers to the dB values from microperimetry. **C** Scatterplot of the mean sensitivities of normal eyes (unfilled) and eyes with cuticular drusen (filled). Group data is summarised by the superimposed box plot. **D** Mean sensitivity is plotted against age for all eyes

Microperimetry results in a 38-year-old eye with cuticular drusen, superimposed on near-infrared autofluorescence image, showed a sensitivity range between 26 and 32 dB, with mean sensitivity (MS) of 29.1 dB (Fig. 2A). In an older, 53-year-old eye, MS was lower at 24.9 dB with range between 23 and 27 dB (Fig. 2B). Mean (SD) (26.7 [2.2] dB) MS in the cuticular drusen cohort straddled the 95% confidence interval from the normal cohort (25.2 to 30.7 dB) (Fig. 2C). MS plotted against age (Fig. 2D) showed that, in the healthy cohort, MS declined by 0.03 dB a year (*y* =  − 0.03*x* + 29.8, *R*^2^ = 0.13, *p* < 0.001). The age-related sensitivity decline was similar in the cuticular drusen cohort (*y* = -0.06*x* + 29.9, *R*^2^ = 0.13, *p* = 0.06).

### Examining pointwise deviation from age- and location-adjusted reference

**Fig. 3:**
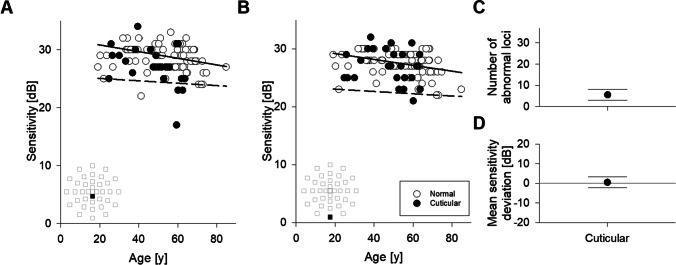
Analysing AMD data using age- and location-adjusted reference. **A**, **B** Examples demonstrating quantile regression. Retinal sensitivity versus age in all healthy eyes (unfilled) is shown for locus next to (**A**) and away from (**B**) the centre of the grid. Sensitivity values were also plotted for cuticular drusen eyes (filled). Quantile regression lines for the median (solid) and 2.5th quantile (dashed) are plotted. Inset shows the location of the loci in the test grid (filled square). **C** Mean number of abnormal loci in cuticular drusen eyes defined using the 2.5th percentile cutoff, horizontal lines indicate 95% CI. **D** Mean sensitivity deviation in cuticular drusen eyes, horizontal lines indicate 95% CI

At each MAIA test locus, retinal sensitivities from all healthy eyes were plotted against their respective age, and quantile regression was utilised to calculate the 2.5th percentile line (Fig. 3A, B; dashed line). Any data point that falls below the 2.5th percentile line was considered having abnormally low sensitivity for the age. The median line (solid) was utilised to calculate sensitivity deviation. Two examples of performing quantile regression using pointwise normative data and the identification of abnormal loci in the cuticular drusen cohort are demonstrated. In the locus closer to the grid centre (Fig. 3A); the proportion of the cuticular drusen cohort that fell below the 2.5th percentile line was 3/27 (11%). In the locus further from the grid centre (Fig. 3B), the proportion of the cuticular drusen eyes that fell below the 2.5th percentile line was 1/27 (4%). Across the entire 37-loci testing grid, the mean (95% CI) number of abnormal loci as defined by the 2.5th percentile cutoff in the cuticular drusen cohort (Fig. 3C) was 5.5 (3.0 to 8.1). Note that the lower limit of the 95% CI was greater than 0. However, the mean sensitivity deviation across the 27 cuticular drusen eyes was not significantly different to 0 dB when analysed across the whole grid (Fig. 3D, 95% CI − 2.3 to + 3.4 dB).

**Fig. 4:**
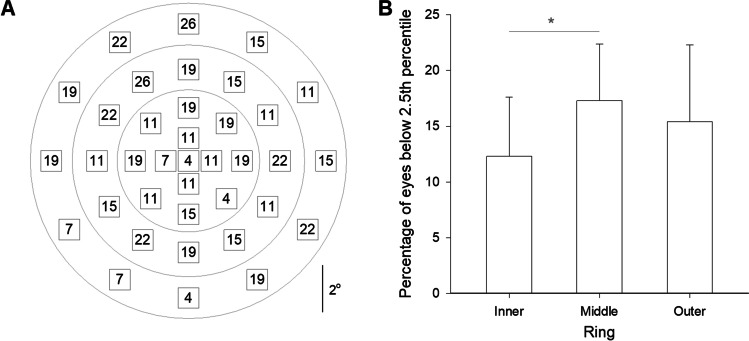
Distribution of loci below age- and location-adjusted cutoff. **A** The percentage of cuticular drusen eyes at each locus fell below the 2.5th percentile cutoff. **C** The test grid is divided into three rings, see grey circles in panel A. The mean percentage of eyes that falls below the 2.5th percentile cutoff at each ring is plotted. Error bars indicate SD

At each locus, the percentage of cuticular drusen eyes that showed sensitivity below the 2.5th percentile using the age- and loci-specific reference is shown (Fig. 4A). The testing grid was divided into three rings containing a similar number of loci (inner: 13, middle: 12, outer: 12; grey rings). Within each ring, the mean (SD) percentage of eyes below the 2.5th percentile cutoff was 12.3 (5.3) %, 17.3 (5.1) %, 15.4 (6.9) % for the inner, middle, and outer rings, respectively. There was a significant difference in the mean percentage of eyes below the 2.5th percentile cutoff between the inner and outer rings (Fig. 4B, p < 0.05).

## Discussion

In this study, detailed phenotyping was performed to precisely identify a cohort of patients with cuticular drusen before investigating functional changes. We then analysed pointwise deviation using an age- and loci-specific reference database. Importantly, our data demonstrated localised defects despite the nonsignificant difference in mean sensitivity between the cuticular drusen cohort and age-matched normals.

Cuticular drusen typically manifest in early adulthood, as opposed to later in life in the classic soft drusen AMD phenotype. Given that younger eyes with cuticular drusen are typically asymptomatic, hence, to the best of our knowledge, there is no data on retinal sensitivity changes due to the disease. At first glance, without accounting for age and test location, our data showed no difference in mean sensitivity between age-matched normal and cuticular drusen eyes. We then explored the cuticular drusen data in further detail using an age- and loci-specific reference threshold and found that, in the 37-loci testing grid, retinal sensitivity in a mean of 5.5 loci (15%) was considered lower than normal. In addition, these loci with reduced sensitivity tended to be distributed further away from the centre of the grid. More importantly, mean sensitivity, which is commonly examined in microperimetry studies, was not sensitive enough to detect the reduction in pointwise sensitivity. A previous study reported an MS deviation of 2.1 dB in eyes with cuticular drusen [7], which differs from our finding of no significant mean sensitivity loss between normal and cuticular drusen eyes. It should be noted that, in the aforementioned study, all patients had the clinical diagnosis of intermediate AMD, and that cuticular drusen were defined as the ‘predominant’ drusen type in the patient’s eye. Hence, it is likely that the presence of soft drusen, which typically coexists in older patients with cuticular drusen [16], may contribute to the reduction of retinal sensitivity [17]. In support of our postulation, our patient cohort was younger (mean [SD]: 48.8 [12.9]) than the aforementioned study (57.9 [8.8]).

It has been shown that mesopic MS in intermediate AMD was lower compared to control eyes [18, 19]. In addition, mesopic retinal sensitivity has been shown to decrease over time in large (> 125 µm) but not small (< 63 µm) or intermediate (63–125 µm) soft drusen using both pointwise [20] and mean [21] sensitivity analysis. However, there have been variable findings regarding mesopic sensitivity change with reticular pseudodrusen. It has been reported that, using multivariate modelling, there was no association between microperimetry MS and reticular pseudodrusen [22]. However, other groups have shown a decrease in MS in reticular pseudodrusen compared to eyes with age-related macular degeneration with no reticular pseudodrusen [23, 24] or normal eyes [25]. We found pointwise, not mean sensitivity change in our cohort, demonstrating the subclinical reduction in macular sensitivity in cuticular drusen and earlier in the disease course of the AMD spectrum.

Although our results illustrate the importance of analysing microperimetry data using age- and loci-specific reference thresholds in order to identify pointwise functional deficits in cuticular drusen, there are several limitations that need to be considered. First, some of the microperimetry testing points may be partially projected onto the drusen. However, the main purpose of the study is not to examine the effect of cuticular drusen on photoreceptor function. The underlying disease in cuticular drusen is in the RPE, and the actual drusen is a clinically detectable sign. Hence, there may be a downstream effect of photoreceptor dysfunction due to RPE abnormalities that are not visible clinically, which could be detected by microperimetry. Second, the test–retest variability was not addressed. In both control and patient eyes, data analysis was performed using the subjects’ first, and often, only test result. In addition, there was no separate training test prior to the actual microperimetry test, which could potentially underestimate retinal sensitivity in both the cuticular cohort and the controls. Future investigation is required to establish the extent of the learning effect in eyes with cuticular drusen. Third, the reference database was established using a mixture of patients with unilateral eye disease and healthy controls instead of prospective recruitment of healthy subjects with no systemic, retinal, and optical abnormality. Despite these limitations, we were able to show locus-specific sensitivity reduction in a cross-section of eyes with cuticular drusen. Another approach to analysing this dataset would be to examine the within-eye variance in sensitivity compared to the normal cohort but a larger sample size would be required to detect significance.

In conclusion, we showed that macular sensitivity in eyes with cuticular drusen only can be reduced when examining pointwise deviation, but mean sensitivity is not an effective endpoint to detect a reduction in sensitivity. Therefore, both in the clinic and in future clinical trials, the pointwise deviation should be assessed in cuticular drusen.

## Supplementary Information

Below is the link to the electronic supplementary material.

Supplementary file1 (DOCX 19 KB)
