# Supplementary material for: Localised relative scotoma in cuticular drusen
Source: Graefes Arch Clin Exp Ophthalmol. 2022 Feb 7;260(7):2157–64. doi: 10.1007/s00417-022-05570-4 (PMC9203397; doi:10.1007/s00417-022-05570-4)
Supplement: Supplementary file 1 — Supplementary file1 (DOCX 19 KB) [file 417_2022_5570_MOESM1_ESM.docx]

**Supplementary Material**

**Localized relative scotoma in cuticular drusen**

Jason Charng, Chandrakumar Balaratnasingam, Mary S Attia, Rachael C. Heath Jeffery, Fred K. Chen

**Supplementary Table S1.** Clinical data for 80 control subjects

**Supplementary Table S2.** Clinical data for 27 cuticular drusen patients

**Supplementary Table S1.** Clinical data for 80 control subjects

| **ID** | **Eye Analysed** | **Age** | **Sex** | **63% BCEA (°²)** | **Fellow Eye Diagnosis**^†^ |
| --- | --- | --- | --- | --- | --- |
| 1 | L | 19.0 | M | 0.4 | Coats disease |
| 2 | R | 20.2 | F | 1.4 | normal |
| 3 | R | 21.9 | F | 1.6 | normal |
| 4 | R | 23.5 | M | 1.6 | naevus |
| 5 | R | 26.7 | F | 0.4 | normal |
| 6 | R | 31.8 | F | 0.7 | normal |
| 7 | L | 33.5 | M | 0.1 | normal |
| 8 | L | 34.1 | F | 1.5 | amblyopia |
| 9 | R | 34.2 | M | 0.5 | central serous retinopathy |
| 10 | R | 35.2 | F | 0.2 | normal |
| 11 | L | 35.2 | M | 0.2 | normal |
| 12 | L | 35.8 | M | 0.5 | normal |
| 13 | R | 36.4 | F | 0.4 | normal |
| 14 | R | 36.8 | M | 0.9 | epiretinal membrane |
| 15 | L | 37.7 | F | 0.4 | central retinal vein occlusion |
| 16 | R | 38.3 | F | 2.1 | normal |
| 17 | L | 39.5 | F | 2.7 | retinal detachment |
| 18 | R | 40.0 | M | 2.5 | normal |
| 19 | R | 40.0 | F | 0.1 | normal |
| 20 | R | 41.1 | F | 0.3 | normal |
| 21 | R | 43.6 | M | 0.6 | normal |
| 22 | R | 44.2 | F | 0.2 | CHRPE |
| 23 | R | 44.3 | F | 1.8 | normal |
| 24 | L | 44.4 | F | 0.2 | choroidal naevus |
| 25 | L | 46.6 | F | 0.1 | pigmented lesion |
| 26 | R | 46.6 | F | 1.8 | normal |
| 27 | R | 47.6 | F | 0.5 | retinoschisis |
| 28 | L | 48.4 | F | 1.5 | peripheral retinal hole |
| 29 | L | 49.0 | M | 1.1 | punctate inner choroidopathy |
| 30 | R | 50.2 | M | 0.5 | rhegmatogenous retinal detachment |
| 31 | R | 50.9 | M | 1 | retinal detachment |
| 32 | R | 51.1 | F | 0.6 | normal |
| 33 | L | 51.5 | M | 1.8 | macular hole |
| 34 | L | 51.6 | F | 0.5 | posterior vitreous detachment |
| 35 | R | 52.0 | F | 0.3 | normal |
| 36 | R | 52.5 | M | 0.7 | normal |
| 37 | L | 53.7 | M | 1 | normal |
| 38 | R | 56.1 | F | 1.7 | osteoma |
| 39 | L | 56.4 | M | 0.5 | melanoma |
| 40 | R | 58.0 | M | 0.2 | melanoma |
| 41 | L | 58.2 | M | 3.8 | retinal detachment |
| 42 | R | 58.3 | M | 0.9 | epiretinal membrane |
| 43 | R | 58.7 | F | 1.6 | naevus |
| 44 | L | 58.8 | F | 0.2 | rhegmatogenous retinal detachment |
| 45 | R | 59.3 | F | 1.2 | retinal tear |
| 46 | R | 59.5 | F | 0.3 | posterior vitreous detachment |
| 47 | R | 59.9 | M | 0.6 | retinal vein occlusion |
| 48 | L | 60.7 | M | 0.8 | posterior vitreous detachment |
| 49 | L | 61.2 | M | 0.7 | rhegmatogenous retinal detachment |
| 50 | L | 61.2 | M | 0.5 | retinoschisis |
| 51 | L | 62.0 | M | 0.4 | choroidal melanoma |
| 52 | R | 62.1 | M | 0.1 | retinal tear |
| 53 | R | 62.4 | F | 4.8 | retinal detachment |
| 54 | R | 62.5 | F | 0.3 | trauma |
| 55 | R | 62.7 | F | 1.7 | normal |
| 56 | R | 62.9 | F | 3.2 | normal |
| 57 | L | 63.5 | F | 2.1 | vitreomacular traction |
| 58 | R | 64.1 | F | 0.3 | epiretinal membrane |
| 59 | R | 64.3 | F | 0.1 | choroidal naevus |
| 60 | R | 64.4 | M | 0.4 | normal |
| 61 | R | 64.7 | M | 1.7 | central serous retinopathy |
| 62 | L | 64.7 | F | 2.2 | epiretinal membrane |
| 63 | R | 64.9 | M | 0.1 | normal |
| 64 | R | 66.0 | F | 0.1 | lamellar hole |
| 65 | L | 66.7 | M | 0.8 | retinal tear |
| 66 | R | 68.0 | F | 0.3 | central serous retinopathy |
| 67 | R | 68.0 | F | 0.5 | branch retinal vein occlusion |
| 68 | L | 68.1 | M | 1.0 | central serous retinopathy |
| 69 | R | 68.1 | M | 0.1 | normal |
| 70 | R | 68.4 | F | 0.3 | amblyopia |
| 71 | R | 68.5 | F | 0.4 | normal |
| 72 | R | 69.0 | M | 0.6 | rhegmatogenous retinal detachment |
| 73 | R | 69.8 | M | 0.6 | epiretinal membrane |
| 74 | L | 70.6 | F | 0.1 | haemorrhage |
| 75 | R | 72.1 | M | 3.5 | epiretinal membrane |
| 76 | L | 72.6 | F | 0.4 | retinal vein occlusion |
| 77 | L | 72.9 | F | 3.7 | retinal detachment |
| 78 | R | 73.1 | F | 1.3 | macular hole |
| 79 | R | 74.7 | M | 0.1 | normal |
| 80 | R | 84.8 | M | 1.5 | epiretinal membrane |

BCEA, bivariate contour ellipse area

**Supplementary Table S2.** Clinical data for 27 cuticular drusen patients

| **ID** | **Eye Analysed** | **Age** | **Sex** | **63% BCEA (°²)** |
| --- | --- | --- | --- | --- |
| 1 | R | 24.7 | F | 3.1 |
| 2 | R | 25.8 | M | 1.3 |
| 3 | R | 26.4 | F | 0.2 |
| 4 | R | 30.0 | M | 0.2 |
| 5 | R | 33.2 | F | 0.2 |
| 6 | R | 36.8 | F | 1.3 |
| 7 | R | 38.3 | M | 0.2 |
| 8 | R | 39.5 | M | 0.3 |
| 9 | L | 46.1 | M | 0.4 |
| 10 | R | 47.4 | F | 0.6 |
| 11 | R | 48.6 | F | 1.3 |
| 12 | R | 48.8 | F | 2.2 |
| 13 | R | 50.1 | F | 2.6 |
| 14 | R | 51.8 | F | 1.8 |
| 15 | R | 52.8 | F | 1.1 |
| 16 | R | 53.8 | F | 1.4 |
| 17 | R | 54.6 | F | 1.0 |
| 18 | R | 55.2 | M | 0.3 |
| 19 | R | 55.7 | F | 0.0 |
| 20 | R | 59.4 | F | 0.3 |
| 21 | R | 59.4 | F | 0.3 |
| 22 | R | 60.2 | F | 0.7 |
| 23 | R | 60.3 | M | 0.1 |
| 24 | L | 61.9 | M | 0.0 |
| 25 | R | 62.5 | F | 2.2 |
| 26 | R | 63.4 | M | 0.4 |
| 27 | R | 63.7 | F | 4.2 |
